# Diversity–disease relationships in natural microscopic nematode communities

**DOI:** 10.1098/rsos.242088

**Published:** 2025-04-02

**Authors:** Robbert van Himbeeck, Jessica N. Sowa, Hala Tamim El Jarkass, Wenjia Wu, Job Oude Vrielink, Joost A. G. Riksen, Aaron Reinke, Lisa van Sluijs

**Affiliations:** ^1^ Laboratory of Nematology, Wageningen University and Research, Wageningen, Gelderland, The Netherlands; ^2^ Department of Biology, West Chester University of Pennsylvania, West Chester, PA, USA; ^3^ Department of Molecular Genetics, University of Toronto, Toronto, Ontario, Canada; ^4^ Guangdong Provincial Key Laboratory of Applied Botany, South China Botanical Garden, Guangzhou, Guangdong, People's Republic of China; ^5^ Key Laboratory of National Forestry and Grassland Administration on Plant Conservation and Utilization in Southern China, South China Botanical Garden, Chinese Academy of Sciences, Guangzhou, Guangdong, People's Republic of China

**Keywords:** biodiversity, diversity–disease effect, metabarcoding, nematodes, parasites, microsporidia

## Abstract

Host diversity can affect parasite prevalence, a phenomenon widely studied in macroscopic organisms. However, data from microscopic communities are lacking, despite their essential role in ecosystem functioning and the unique experimental opportunities microscopic organisms offer. Here, we study diversity–disease effects in wild nematode communities by profiting from the molecular tools available in the well-studied model nematode *Caenorhabditis elegans*. Nanopore sequencing was used to characterize nematode community diversity and composition, whereas parasites were identified using nine distinct experimental assays based on fluorescent staining or fluorescent reporter strains. Our results indicate that biotic stress is abundant in wild nematode communities. Moreover, in two assays, diversity–disease relations were observed: microsporidia and immune system activation were more often detected in relatively species-poor communities. Other assays, targeting different parasites, were without diversity–disease relations. Together, this study provides the first demonstration of diversity–disease effects in microbial communities and establishes the use of nematode communities as model systems to study disease–diversity relationships.

## 1. Introduction

Ecosystem functioning depends on biodiversity, which is shaped by parasites [1–3]. Increased (host) biodiversity can theoretically both cause increased (i.e. amplification effect) or decreased (i.e. dilution effect) parasite prevalence [2,4,5]. Generally, amplification effects occur when the parasite becomes more prevalent due to an increased availability of suitable hosts. Dilution effects result from reduced parasite reproduction because parasites experience difficulties finding a high-quality host among all species present. In contrast, parasites that replicate in a low-biodiversity population spread easily because of the high chances that the next organism they encounter is also a highly suitable host. Host–parasite relationships have been primarily studied in macroscopic species (e.g. [6–8]), despite the necessity of well-functioning microscopic communities for ecosystem stability [3], natural host–parasite interactions in microscopic communities are little understood.

Interpretation of diversity–disease correlations has not always been straightforward, as they depend on complex interactions within the natural community. Diversity–disease effects can be obscured or enhanced by parasite encounters, susceptibility and transmission of the species in the community, the order, timeframe and magnitude in which species enter or disappear from the community, life-history traits of the host and host range of the parasite [4,7–21]. Interestingly, microscopic species that are relatively easy to grow and manipulate would present excellent models for studying diversity–disease effects, in particular, when information from laboratory experiments can be combined with field observations. A model system where complex natural observations can be paired with controlled experiments will provide new experimental opportunities and biological insight into diversity–disease relations of (micro)organisms.

Nematodes present one of the most diverse and abundant groups of microscopic organisms worldwide [22], and some species possess characteristics that make them excellent model microscopic organisms. Of all nematodes, the free-living bacterivorous nematode *Caenorhabditis elegans* is a key model organism in many biological fields, ranging from developmental biology to neuroscience [23–26]. Today’s science not only focuses on the molecular and genetic understanding of this 1 mm sized organism in the laboratory, but also considers its natural ecology, which led to the discovery of many naturally infecting parasites [27]. Parasites infecting *C. elegans* and related bacterivorous species comprise opportunistic bacteria and obligatory parasites including oomycetes, microsporidia and viruses, many of which cause deadly infections [27–32]. Recently, complete sequencing of the 18S gene using long-read technology of Nanopore was established as a technique to determine nematode community diversity and composition in large-scale experiments [33]. This enables high-resolution nematode community characterization, thereby providing a new opportunity to study diversity–disease interaction in natural communities of the model nematode *C. elegans* [33].

The aim of this study was to apply the nematode *C. elegans* as a model species for investigating diversity–disease interactions in microscopic communities. We hypothesized that (i) *C. elegans* is a suitable diversity–disease model organism, as we expect that because of its opportunistic nature, it will occur in various nematode communities, and bacterial, microsporidian and viral parasites can be extracted from wild populations; and (ii) disease–diversity relationships also occur in microscopic communities. To test our hypotheses, we set three research objectives. First, we tested the similarity in the occurrence of *C. elegans* across ephemeral nematode communities with varying richness and species composition by reconstructing the nematode diversity and community composition using 18S amplicon sequencing. Second, we determined the prevalence of parasites of nematodes within ephemeral nematode communities using nine assays of parasite identification. Third, we determined to what extent nematode (i.e. hosts) community diversity associates with parasite prevalence. Together, our results provide the first indication of disease–diversity relationships within microscopic communities and prospect a new experimental model system.

## 2. Methods

### 2.1. Sample collection and nematode isolation

**Figure 1.**
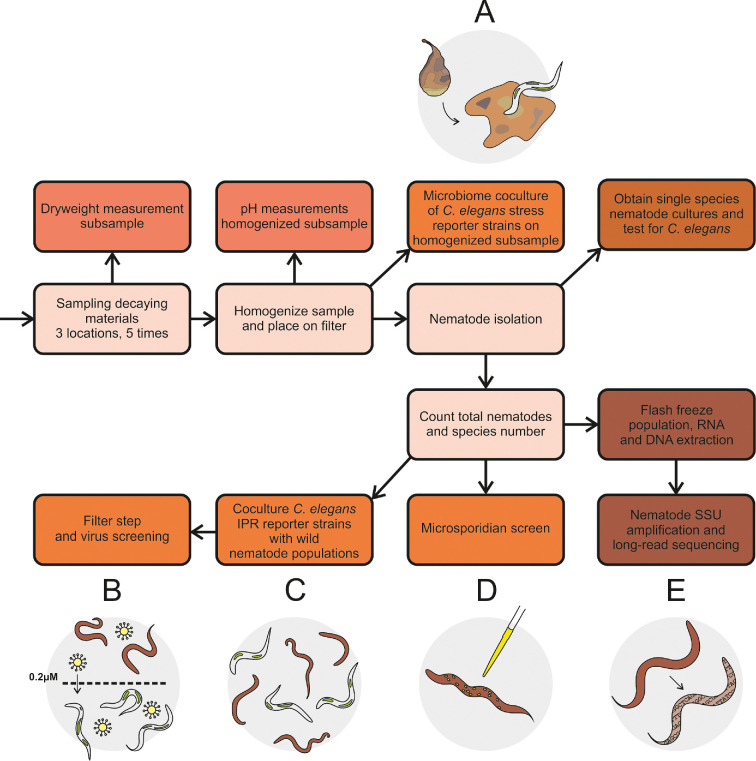
Experimental workflow of the overall study—Nematodes were collected from three different sample sites on five separate occasions in autumn. Of these, the dry weight and pH were determined. After the nematode populations were isolated, individual nematodes were singled out and, when applicable, tested for the presence of *Caenorhabditis elegans*. Four methods were applied to screen for biotic stresses in the isolated communities: (A) co-culturing of reporter strains with the homogenized substrate, (B) co-culturing *C. elegans* reporter strains on 0.22 µm filtrate of the isolated nematode community for virus detection, (C) co-culturing of a subsample of the isolated nematodes with reporter strains and (D) direct staining of a subsample of the isolated nematodes to visualize microsporidia. Finally, the remaining nematodes were flash frozen to extract RNA and DNA. The nematode small subunit ribosomal ribonucleic acid (SSU rRNA) in these samples was polymerase chain reaction (PCR) amplified, and long-read sequencing was performed to identify nematode species present in the samples (E).

Sampling was performed five times during autumn at the following three locations in The Netherlands: private garden (Heelsum), private vegetable garden (Wageningen) and patch of green surrounding a ditch (Renkum) (electronic supplementary material, table S1 and figures S1 and S2). The active nematode community was isolated from the plant samples as previously described [34]. Additionally, details and a schematic overview covering the sample collection and nematode isolation are in electronic supplementary material, text S1 and figure 1.

### 2.2. Nematode community characterization using nanopore sequencing

DNA and RNA were extracted from samples that contained at least 100 nematodes at the moment of flash-freezing. From the 112 samples that met this requirement, RNA and DNA were extracted as described previously by Harkes *et al*. [35]. In short, nematodes were bead homogenized using 3 mm beads using a Tissuelyser II (Qiagen). Then, RNA was extracted with a pH 4.5 phenol washing step, followed by a DNA isolation buffer washing step at pH 8.0. DNA and RNA concentrations were determined via Qubit fluorometer measurements (Thermo Scientific). After DNA/RNA extractions, 81 samples contained sufficient DNA quantity for 18S rRNA amplification. Nematode communities were characterized using the workflow by van Himbeeck *et al.* [33], with minor adjustments [33]. EXP-NBD196 (Oxford Nanopore Technologies Ltd, UK) barcoded primers 988F (5′-ctcaaagattaagccatgc-3′) and 2646R (5′-gctaccttgttacgactttt-3′) were used to amplify the near-complete 18S rRNA fragment (approx. 1750 bp) [33,36,37]. Polymerase chain reaction (PCR) was performed in quadruplicate per sample, and each reaction consisted of 12.5 µl LongAMP Taq 2 × MM, 400 nM of each primer, 3 µl DNA template and 5.5 µl autoclaved Milli-Q water. After amplification, all four PCR replicates were pooled, and DNA amplification was verified on agarose gels. Finally, DNA concentrations were determined via Qubit 4 fluorometer measurements (Thermo Scientific).

The 18S rRNA was successfully amplified for 79 samples that were used for sequencing in four batches, where each library contained equimolar ratios of all samples. Unwanted small fragments (less than 600 bp) were removed prior to sequencing using NucleoMag NGS beads (0.5 : 1 bead : sample ratio) (Macherey Nagel). Library preparation was performed using the SQK-LSK112 kit, following the instructions of the manufacturer. Sequencing was performed on a MinION Mk1C using R9.4.1 flow cells.

Base-calling was performed using Guppy (v. 6.2.1) in super-accuracy mode (Oxford Nanopore Technologies PlC., UK). The base-called reads were demultiplexed using Guppy barcoder (v. 6.2.1) and adapters and barcodes were removed. Three samples (LvS26, LvS81 and LvS148) were excluded from the analysis at this stage because insufficient reads were obtained. Basecalling quality was assessed using NanoPlot (v. 1.40.0) (mean Phred score greater than 15) and Decona (v. 0.1.3) was used to filter 1400−2600 bp reads with a greater than Q15 quality score [38,39]. Decona then clustered reads at 95% identity, and polished Medaka consensus sequences were created from clusters containing at least 100 reads. NCBI BLASTn (National Center for Biotechnology Information Nucleotide Basic Local Alignment Search Tool) was used with an in-house database for taxonomic identification of nematode species [33,36]. Identifications with a similarity below 97% were excluded from the dataset. This data processing resulted in an Operational Taxonomic Unit (OTU) table containing the number of sequencing reads of each nematode taxon per sample. From this table, the number of species per sample was determined by summing the number of taxa with a read count larger than 0.

### 2.3. Parasite screening in the homogenized substrate using fluorescent reporter strains

The presence of parasites in the sampled substrate was first explored by growing *C. elegans* reporter strains in a subsample of the homogenized substrate (for details, see electronic supplementary material, text S1). *Caenorhabditis elegans* reporter strains AGD926 (zcIs4[*hsp-4::GFP*]), SJ4100 (zcIs13[*hsp-6p::GFP*]) and AU133 (agIs17[*myo-2p::mCherry+irg-1p::GFP*]) were obtained from the Caenorhabditis Genetic Center (CGC). These strains fluoresce when *C. elegans* (i.e. the host) is infected, indicating endoplasmic reticulum (ER) stress, mitochondrial stress or bacterial virulence respectively (electronic supplementary material, table S2) [40] and thus indicate stress due to the microbiota of the homogenized substrate.

Starved populations from the reporter strains (AGD926, SJ4100, AU133) were transferred to fresh 6 cm Nematode Growth Medium (NGM) plates containing *Escherichia coli* OP50 just before nematode isolation from natural substrates. From each homogenized substrate, 200 µl blender solution was equally spread over the plates containing the reporter strains that were then incubated at 20°C for 48 h. After incubation, reporter nematodes exposed to blender solution were checked to investigate if GFP fluorescence was higher than GFP expressed by nematodes grown on control plates containing only *E. coli* OP50 [40]. Fluorescence was checked using an Olympus SZX10 microscope with a NIGHTSEA microscope fluorescence adapter.

### 2.4. Observation of intracellular parasites using intracellular pathogen response and viral stress reporter strains

The remainder of the homogenized substrate (approx. 100 ml) was used to isolate nematode communities (figure 1, electronic supplementary material, text S1). After isolation, nematode communities were co-cultured with each of the following four reporter strains to observe activation of antiparasitic and antiviral pathways: SOW1 and SOW6, which detect upregulation of intracellular pathogen response (IPR) genes *eol-1* and *pals-5,* and SX2790 and SX2999, which detect upregulation of the antiviral gene *lys-3* (electronic supplementary material, table S2) [41]. In some cases, the isolated nematode community did not grow any more, and therefore co-culture was not successful (labelled as ‘co-culture unsuccessful’). Fluorescence was checked once per day for 7 days using an Olympus SZX10 stereomicroscope equipped with an SZX-RFA stereo fluorescence illuminator unit and filter sets for Green Fluorescent Protein (GFP) and Red Fluorescent Protein(RFP) fluorescence viewing. Co-cultures were counted as positive if greater than 10% of the reporter *C. elegans* were fluorescing.

### 2.5. Screening for potential viruses

Co-cultures that resulted in reporter *C. elegans* fluorescence were washed off of test plates using M9 buffer and concentrated into 1 ml total volume via centrifugation. They were then homogenized via vortexing for 4 min with 30−50 (1 mm diameter) silicon carbide beads (BioSpec Products). Homogenate was centrifuged to pellet debris, and supernatant was passed through a 0.22 µm pore size syringe filter (GenClone). Filtrates were seeded onto plates with SOW1, SOW6, SX2790 or SX2999 reporter strains and observed for fluorescence each day for 7 days.

### 2.6. Microsporidian identification in isolated nematode communities

Isolated nematodes were propagated for approximately 5 days on a 6 cm NGM plate seeded with 10× *E. coli* OP50. Cultures that perished before screening were excluded (labelled ‘not screened’). Nematode communities that perished before this procedure or contained mites were not considered in subsequent analyses. Once the population was composed of a large quantity of non-starved adults, a portion of the plate was chunked onto a newly seeded 6 cm NGM plate to continually propagate potential infections. The remainder of the plate was then washed with 700 µl of M9 media and placed in a microcentrifuge tube. Samples were allowed to gravity settle for approximately 1–5 min to remove contaminating bacteria and fungi through supernatant removal. A total of 700 µl of acetone was added to the nematode pellet before spinning down in a centrifuge for 30 s at 8000 relative centrifugal force (rcf). The supernatant was discarded, and 500 µl of the chitin binding Direct Yellow 96 (DY96) solution (1× PBST, 0.1% SDS, 20 µg ml^−1^ DY96) was added. The samples were incubated for 30 min in the dark at 20°C before spinning down in a centrifuge for 30 s at 8000 rcf. The supernatant was removed, and 20 µl of EverBrite Mounting Medium (Biotium) was added to the samples prior to mounting on glass slides for imaging using an Axio Imager.M2 (Zeiss). Z-stacks were captured at 63× using an apotome unit with maximum projection. Samples were considered infected with microsporidia if spore clusters were visible in a population. Spore sizes were assessed by measuring the length and width of at least 45 spores using Zen software.

To identify the species of microsporidia present within a nematode sample, molecular characterization was performed. Briefly, 20 large adult nematodes were placed in 10 µl of lysis buffer (50 mM KCl, 100 mM Tris–HCl, 2.5 mM MgCl_2_, 0.45% NP40) and in a thermocycler at 65°C for 60 min, followed by 95°C for 15 min. A total of 2 µl of lysate was then used as template in a PCR reaction with the forward primer V1F [5′-CACCAGGTTGATTCTGCCTGAC-3′] and the reverse primer 1492 r [5′-GGTTACCTTGTTACGACTT-3′] or 18sR1492 [5′-GGAAACCTTGTTACGACTT-3′] to amplify microsporidian 18S. Sanger sequencing was performed on amplicons using the same primers listed above. Host density and frequency were subsequently compared with microsporidian presence. Host density and frequency were inferred from metabarcoding data, where host density was defined as the number of individuals of a certain host species per wet gram of substrate. Host frequency was defined as the proportion of the host species among the different nematode species in the sample.

### 2.7. Data analysis

All data were processed and visualized using the package *tidyverse* and *ggpubr* in custom written scripts in R (v. 4.2.1) [42,43]. Generalized linear models (glm), Chi-square and Wilcoxon rank sum tests were computed with *R base*. The effect of factors on community compositions was tested after creating a ‘physeq’ object in the *phyloseq* package (v. 1.44.0) and performing a PERMANOVA based on Bray–Curtis dissimilarity index (*n* = 10 000) using ‘adonis2’ from the *vegan* package (v. 2.6.4) [44,45]. The *vegan* package was also used for calculating alpha diversity indices (richness and Shannon index (*H*′)) with ‘specnumber’ and ‘diversity’ functions [44]. The nematode richness was normalized (for substrate and community size) to facilitate cross-sample comparisons as the samples collected in this study varied because of the ephemeral nature of the substrates and the consequent opportunistic sampling method that was applied. Therefore, normalized nematode richness was defined as the total number of nematode species (based on sequencing data) found in a sample, divided by the number of individuals (based on microscopy-based count data) per gram wet substrate found in that sample.

## 3. Results

### 3.1. Community composition and richness with and without *Caenorhabditis elegans* presence

**Figure 2.**
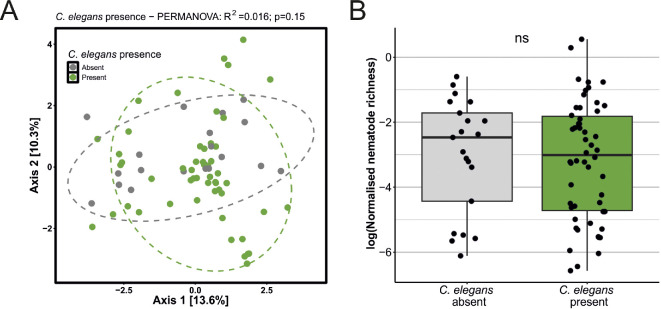
Community composition and richness of ephemeral nematode communities, where *Caenorhabditis elegans* is absent or present. (A) PCoA plot based on robust Aitchison distance for communities where *C. elegans* is present (green) or absent (grey). (B) Nematode richness (in normalized species richness) in samples where *C. elegans* was absent or present. ns, not significant.

To be able to use *C. elegans* as a model for studying diversity–disease effects, it is necessary to verify this species occurs in nematode communities with varying richness (i.e. alpha diversity) and differences in composition (i.e. beta diversity). Ephemeral nematode communities were therefore characterized through sequencing, which identified 112 nematode taxa, primarily consisting of enrichment opportunists (i.e. r-strategists, cp-1) and basal fauna (cp-2) (electronic supplementary material, tables S3−S6) [46]. Communities where *C. elegans* was present or absent (figure 2A) did not separate in the PCoA plot based on the robust Aitchison distance (PERMANOVA, *p* = 0.15, *R*^2^ = 0.016). Furthermore, no differences in (normalized) richness could be observed between communities containing or excluding *C. elegans* (Wilcoxon rank-sum test, *p* = 0.73; figure 2B; electronic supplementary material, table S7). Together, these findings show that *C. elegans* presence in ephemeral communities is not dependent on the community richness or composition.

### 3.2. Parasite prevalence in ephemeral nematode communities

**Figure 3.**
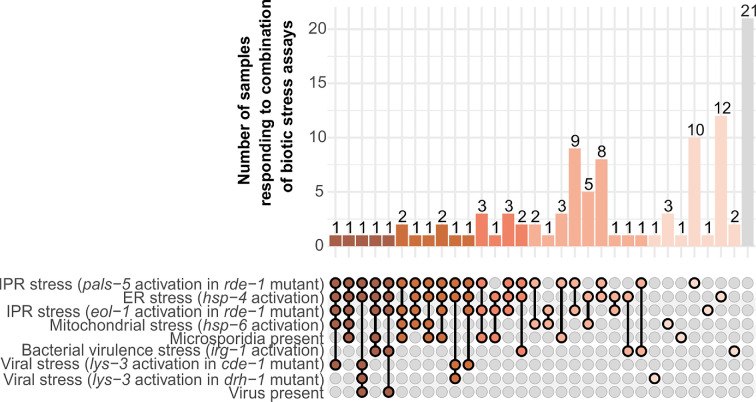
Presence of parasites in natural nematode communities—Overlap of biotic stress indications within each of the samples. The plot shows how many samples (count *y*-axis) responded to specific combinations of biotic stress assays (*x*-axis). Colours illustrate the number of stress assays for a sample was found positive ranging from five (red) to none (grey).

The prevalence of parasites in ephemeral nematode communities was examined by screening the collected nematode communities for phenotypes that indicate (i) the presence of microsporidia, (ii) mitochondrial stress, ER stress and stress caused by bacterial virulence [40], (iii) IPR activation, and (iv) presumptive viruses (figure 1). Some nematode communities died before they could be screened or could not be maintained successfully in the laboratory and were excluded from further analysis. Overall, 80% of the collected samples responded in at least one of the experimental assays, and in most cases, samples were found positive for multiple stressors (figure 3, electronic supplementary material, figure S2).

### 3.3. Nematode community diversity as driver of parasite prevalence

**Figure 4.**
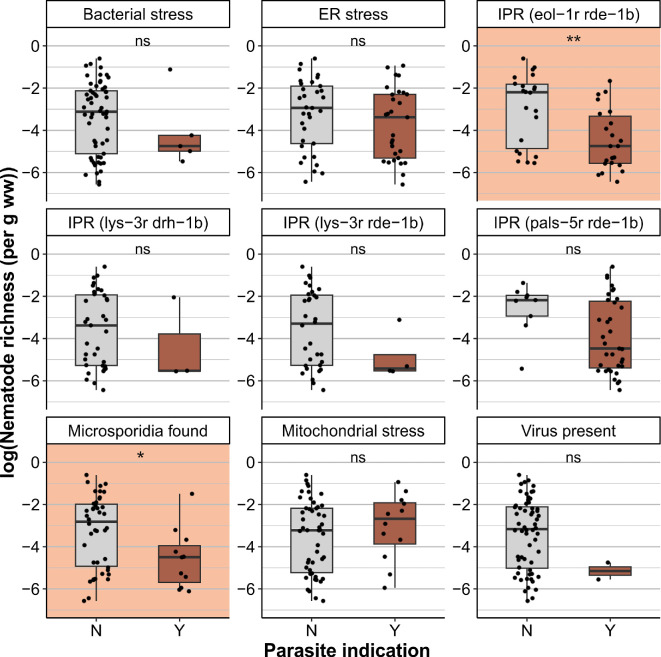
Richness–disease relationships in natural nematode communities. Sequence-based normalized richness for each of the nine performed screenings that indicate parasite presence in wild nematode populations. Plots indicate if samples did (Y) or did not (N) indicate bacterial stress (upregulation of the *Caenorhabditis elegans* gene *irg-1*), ER stress (upregulation of the *C. elegans* gene *hsp-4*), mitochondrial stress (upregulation of the *C. elegans* gene *hsp-6*) or IPR stress (by upregulation of the *C. elegans* genes *pals*-5, *eol-1* or *lys*-3) or presence of viruses or microsporidia. Assays in which significant differences were found (Wilcoxon, *p* < 0.05) are highlighted in orange. ‘ns’ p > 0.05, ‘*’ p < 0.05, ‘**’ p < 0.01

Parasite presence was then combined with nematode diversity for each of the experimental assays to assess if community diversity is linked to parasite prevalence. For two assays, diversity–disease effects were observed: (i) microsporidia were more often present in communities with lowered nematode richness and (ii) activation of the *eol-1*-based IPR reporter strain was more often observed in co-cultures when communities had a lowered nematode richness (figure 4). For the other experimental assays, we did not observe diversity–disease relationships (figure 4, electronic supplementary material, figure S3). Because we obtained the most detailed data (incl. host species) for the microsporidia species (electronic supplementary material, table S8), we further looked into this data to discover potential drivers and confounding factors of diversity–disease effects.

**Figure 5.**
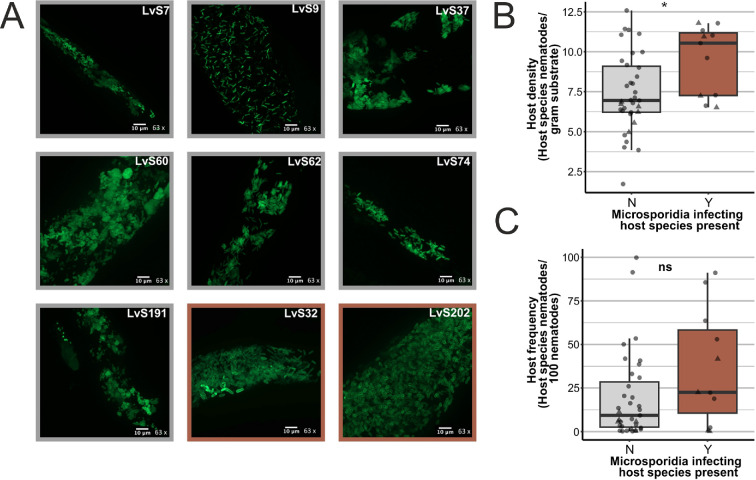
Host density and frequency in microsporidia-infected nematode populations. (A) Samples carrying microsporidia that infect *Caenorhabditis elegans* (grey box) and *P. uniformis* (brown box) were analysed for host density and frequency within the nematode community. (B) Host density of *C. elegans* (circles) and *P. uniformis* (triangles) with (Y) or without (N) microsporidia discovered. (C) Host frequency of *C. elegans* (circles) and *P. uniformis* (triangles) with (Y) or without (N) microsporidia discovered.

Microsporidia were morphologically identified in 15 out of 76 screened samples (16%) (electronic supplementary material, figures S4 and S5). Almost half of these microsporidia species were found in *C. elegans* (electronic supplementary material, table S8). The microsporidia species included the previously described species *Nematocida parisii* [28,47], but most microsporidia could not be molecularly identified and may represent novel species as the primers could not amplify the DNA of these species. Moreover, microsporidia were identified in *Pristionchus uniformis*, *Panagrolaimus rigidus* and in other nematodes of which the species could not be determined. Microsporidia spores are resistant to environmental conditions, including pH and desiccation [48,49], and these factors might also affect nematode communities. In this study, microsporidia were more often observed on alkaline substrates (pH can influence the infectivity of microsporidia [50]), whereas substrate moisture varied (electronic supplementary material, figure S6). Nevertheless, pH and nematode richness were not correlated (glm, *p* = 0.46). Therefore, the relation between nematode richness and microsporidian occurrence was explored further in terms of host density and frequency, because diversity–disease relationships can be driven by host density, frequency or a combination of both [4,7,13,14]. Host frequency and density data for *C. elegans* and *P. uniformis* were selected, because for these species metabarcoding data were available for the samples in which microsporidia were identified (figure 5A). Host density (*p* = 0.016, figure 5B), but not host frequency (*p* = 0.15, figure 5C) related to microsporidian presence in these nematodes, although additional data would be essential to observe if the same trends remain across multiple species.

## 4. Discussion

Biodiversity is important as it not only contributes to ecosystem functioning but also to ecosystem stability, as exemplified by the diversity–disease relationships. Although microscopic communities are vital for ecosystem functioning and stability, host–parasite relationships and the diversity–disease relationships have been poorly studied in microscopic communities. Below-ground microbial communities—such as nematode communities—are vulnerable to shifts and biodiversity loss due to global change [51]. We argue that understanding diversity–disease effects in microbial communities is essential for predicting their response to a decrease in biodiversity in terms of parasite dynamics and ecosystem functioning in a rapidly changing environment. Here, we used wild nematode communities to study the effect of host richness on the occurrence of parasites in microbial communities. Nanopore sequencing was applied for high-resolution characterization of nematodes present within each community. With this method, we molecularly defined nematode communities naturally occurring with the important model organism *C. elegans*. Fluorescent reporter tools available in this model organism were used to identify bacterial-, microsporidian- and viral-induced stress inflicted on *C. elegans*. These screenings also discovered potentially new parasite species. In line with the dilution theory, some specialistic parasites were more often found in communities with fewer nematode species, indicating that diversity–disease effects can be found among microscopic organisms.

### 4.1. *Caenorhabditis elegans* as model organism for disease–diversity studies in microscopic communities

Our first research objective addressed the differences in community composition and richness of ephemeral nematode communities, where *C. elegans* is absent or present. Arguably, contrasts in composition and richness between these communities could jeopardize the role of *C. elegans* as model organism for disease–diversity studies in microscopic communities. We observed no differences in community composition and richness between communities with *C. elegans* and those without, supporting our use of *C. elegans* as model species for ephemeral communities without unintentional bias by nematode community composition and species richness.

To efficiently examine diversity–disease effects, we designed a new approach where we utilized reporter strains to visualize stress responses for indirect screening of *C. elegans* bacterial and intracellular parasites. This approach facilitates indirect study of microscopic parasites of *C. elegans*, but does not provide definite evidence of parasite presence or parasite numbers. Nevertheless, in all but one (where co-cultures with reporter strains were unsuccessful) communities where microsporidia were identified via a direct screening, activation of *C. elegans* reporter strains was also noted, suggesting a combination of reporter strains is sufficient to observe parasite presence. This experimental approach thus offers the advantage of studying multiple parasites in the same assay; experimental models to study complex multi-species interactions are so far still relatively limited [52–55].

Each assay used in this study offers distinct strengths, along with possible limitations. The advantage of the microsporidian assay is that it provides the most direct output: microsporidia are readily visualized in all nematode species in the population [56]. This results in a detailed dataset that allows for additional analyses like the ones we have performed for density and frequency correlations. Because the nematodes are fixed when visualized, they cannot be used in following experiments or individually followed over time, in contrast to fluorescent reporter strains of *C. elegans* that can be looked at non-invasively directly on the Petri dish. These screenings by fluorescent reporter strains of *C. elegans* demonstrated their use in this and other studies for assessing the presence of parasites indirectly. *Caenorhabditis elegans* strains carrying reporter genes have been successfully used to assess the pathogenicity of bacterial species [40,57], microsporidia [58,59], oomycetes [29] and viruses [41]. Moreover, strains AGD926, SJ4100 and AU133 are readily available from the CGC, facilitating reproducibility in other studies, and have been used to map the presumed effect of the bacteria within the microbiome of *C. elegans* [40]. The fluorescent strains based on functioning of the IPR respond to a narrower range of parasites than the latter, providing the benefit that they are more specific. Most natural parasites of *C. elegans* were discovered during the last 15 years (e.g. the first virus was the Orsay virus in 2011 [60] and the first microsporidian parasite *N. parisii* was discovered in 2008 [47]) and as a result, the host range of most parasites is not fully investigated yet. This is currently a limitation of the *C. elegans* fluorescent reporter strains as they may or may not detect parasites infecting other nematode species. Future studies focusing on the host range of nematode parasites (such as [61]) will enrich the information obtained by the use of fluorescent reporter strains. Applying fluorescent reporter strains demonstrated that they can respond to stress within a matter of hours, but what remains less clear is how long it takes for fluorescence to become lost after the infection has been cleared. Elongated activation of reporter genes may give false positive results. Here, species richness was determined soon after nematode isolation, and reporter strain activation was subsequently determined based on co-culturing wild and reporter strain nematodes. Hence, reporter strain activation in this study represents a binary output, whereas intensity or timing of infection might also reflect diversity–disease relationships in microscopic communities [62]. Future studies may provide a better understanding of pathogen intensity if (de)activation times of reporter strains could be related to parasite numbers and screening would be performed directly after nematode isolation. Thus, based on our results, fluorescent reporter strains present a crude, yet reliable estimate for detection of infections, whereas direct screening like applied for detecting microsporidia can provide additional benefits such as visualization and quantification of parasites. What assay is chosen for the best results thus depends on the biological question, target parasite and the available resources (time and possibilities for follow-up experiments).

### 4.2. Parasites are prevalent in ephemeral nematode communities

Our second research objective addressed the prevalence of parasites in natural nematode communities. Parasite prevalence within the nematode communities was tested via nine different biotic stress assays. The vast majority of nematode communities in the collected samples responded in at least one of these tests, and often responses to multiple stressors were observed. Individual bacterial species isolated from the habitat of *C. elegans* harmed nematode growth in 22% of the cases. In this study, the *irg-1* bacterial reporter strain was activated in 8% of the samples. These numbers may be lower in this study because nematodes were presented as a mixture of bacteria that they may evade [40] and not all pathogenic bacteria may activate *irg-1*. In agreement with previous studies, we found that presumptive *C. elegans*-infecting viruses were rare and observed in only 2 (1.5%) of the screened communities [31]. Notably, our results potentially under-represent the total number of parasites occurring in the wild, as not all may infect the reporter strains used, parasites might be lost during the isolation procedure and nematodes potentially evade parasites in some of the assays [63–65]. Altogether, these results indicate that biotic stress is frequent in wild nematode populations and suggest that biotic stress is a common burden for wild nematodes.

### 4.3. Nematode community diversity affects parasite prevalence

Our third research objective focused on discovering diversity–disease effects in nematode communities. Within the screen nematode communities, we found effects between nematode host richness and intracellular parasite prevalence as (i) samples containing microsporidian parasites had a lower nematode richness and (ii) the *eol-1-*based intracellular pathogen response reporter strain more often responded to communities with a lower richness. The experimental assays to indicate biotic stress covered diverse possible origins of stress-inducing parasites with varying host specificities. Parasites most prone to experience dilution effects are those that have a narrow host range [13]. Our data correspond with this theory, as we observed potential dilution effects in microsporidian infected communities. Microsporidia are parasites of most types of animals with the vast majority of species only observed to display host and tissue specificity in one or two closely related hosts [50,66,67]. Microsporidia that infect nematodes also often display specialized tissue specificity, though there are examples of more generalist species [28,68]. Microsporidian occurrence in communities with lower species richness thus enhances their chance of encountering a high-quality host. Nevertheless, the often pathogenic or potentially lethal nature of microsporidia may also explain lower nematode richness in communities suffering from microsporidia with a broad host range [69].

Potential dilution effects were also observed using the *eol-1* reporter strain in *rde-1* immunocompromised *C. elegans. Eol-1* is an RNA decapping enzyme that is upregulated during viral and microsporidian infections and is involved in activating enhanced RNAi in response to mitochondrial stress [70–73]. We hypothesize that the ephemeral communities consisting mostly of bacterial-feeding r-strategists could experience strong competition for food in their temporary environment. Competition between species can drive dilution effects, especially in the absence of predation [4]. Although several predatory species were observed, these were often present in low numbers. An exception was formed for facultative predators like *Pristionchus* [74], but these may also feed on bacteria in this habitat. Yet of note, potential predation by other species, such as mites or fungi was not studied here. Moreover, migration of ephemeral-specialized nematodes provides a spatial factor in the host–parasite dynamics that might be compared with that of a fragmented landscape. Fragmentation of ephemeral nematode communities may further contribute to the observation of diversity–disease effects [15,20,75,76]. For other biotic stress reporter assays, we did not observe diversity–disease effects. The *hsp*-6 reporter strain reports mitochondrial stress just like *eol*-1, but this reporter strain lacked the sensitivity-increasing *rde*-1 mutation, which is a possible explanation for no different observations between these two reporter strains. Also, reporter strains (based on *lys*-3) that respond to viruses did not link to nematode richness, despite the narrow host range of viruses [60]. Both communities where viruses were identified contained lower than average nematode richness (figure 4), but the low incidence of viral infections obstructs drawing solid conclusions from this observation. Another of the reporter strains responding to intracellular parasites (based on *pals*-5) did not show any diversity–disease relationship. It was noted that there were relatively few sequenced communities for which expression of this reporter strain was not observed. Finally, bacteria are usually opportunistic parasites not bound to certain hosts; therefore, dilution effects were not expected, but amplification effects may occur. Nevertheless, these were not observed in the reporters strain that indicates bacterial virulence and also not in the ER stress reporter strain that detects stress caused by both generalists and specialists.

### 4.4. Conclusions and future outlook

Our study provides the first demonstration of diversity–disease relationships in microscopic nematode communities. We validated the choice of *C. elegans* as a model system by showing that community biases in host richness are absent. Our novel approach, consisting of multiple stress reporter assays, then revealed that biotic stress is prevalent in ephemeral nematode communities. Finally, we show that the prevalence of some of these pathogens is associated with host diversity, thereby demonstrating diversity–disease relationships in microscopic communities.

Diversity–disease relationships depend on dynamic multi-factorial processes in communities and as such may be strikingly difficult to understand or capture. Studying diversity–disease effects in nematode populations poses a promising outlook for the future. In this study, we have isolated communities from the field through the use of a small plastic bag and have shown these could be observed and subsequently disassembled in the laboratory. Many collected species were successfully cultured afterwards, indicating follow-up experiments can be used to determine exactly which factors could cause diverse–disease effects to occur in nematode communities. Under laboratory settings, one can deduct or control factors, such as species presence, density and select community members (including competitors and predators) based on specific life-history traits. Furthermore, nematode parasites can be examined to trace infection and transmission (even in real time) [61,77]. Together, our results invite further characterization of host–parasite interactions in microscopic communities and open opportunities for using nematode communities as a diversity–disease model system in the laboratory and field.

## Data Availability

Caenorhabditis elegans strains isolated in this study are available from CaeNDR (caendr.org) [78,79]. Custom written scripts and raw data files can be found on [80]. The nematode sequencing data is accessible on Sequence Read Archive (SRA) under BioProject PRJNA1021795. Processed data is included in the Supplementary Tables of this manuscript. Microsporidia 18S sequences are available in the National Center for Biotechnology Information (NCBI) under the following accession numbers: OR636101, OR636102 and OR636103. Supplementary material is available online [81].
